# International consensus for neuroblastoma molecular diagnostics: report from the International Neuroblastoma Risk Group (INRG) Biology Committee

**DOI:** 10.1038/sj.bjc.6605014

**Published:** 2009-04-28

**Authors:** P F Ambros, I M Ambros, G M Brodeur, M Haber, J Khan, A Nakagawara, G Schleiermacher, F Speleman, R Spitz, W B London, S L Cohn, A D J Pearson, J M Maris

**Affiliations:** 1 CCRI, Children's Cancer Research Institute, Vienna, Austria; 2 Center for Childhood Cancer Research, Children's Hospital of Philadelphia, University of Pennsylvania School of Medicine, PA, USA; 3 Children's Cancer Institute Australia, Sydney, Australia; 4 National Cancer Institute, Bethesda, MD, USA; 5 Chiba Cancer Center Research Institute, Japan; 6 Institut Curie, Paris, France; 7 Centre for Medical Genetics, Ghent, Belgium; 8 University of Cologne, Germany; 9 Children's Oncology Group Statistics and Data Center, University of Florida, Gainesville, FL, USA; 10 The University of Chicago, Chicago, IL, USA; 11 Section of Paediatrics, Institute of Cancer Research and Royal Marsden Hospital, Surrey, UK

**Keywords:** neuroblastoma, treatment planning, genomic, translational, international consensus, INRG, genetic risk factors

## Abstract

Neuroblastoma serves as a paradigm for utilising tumour genomic data for determining patient prognosis and treatment allocation. However, before the establishment of the International Neuroblastoma Risk Group (INRG) Task Force in 2004, international consensus on markers, methodology, and data interpretation did not exist, compromising the reliability of decisive genetic markers and inhibiting translational research efforts. The objectives of the INRG Biology Committee were to identify highly prognostic genetic aberrations to be included in the new INRG risk classification schema and to develop precise definitions, decisive biomarkers, and technique standardisation. The review of the INRG database (*n*=8800 patients) by the INRG Task Force finally enabled the identification of the most significant neuroblastoma biomarkers. In addition, the Biology Committee compared the standard operating procedures of different cooperative groups to arrive at international consensus for methodology, nomenclature, and future directions. Consensus was reached to include *MYCN* status, 11q23 allelic status, and ploidy in the INRG classification system on the basis of an evidence-based review of the INRG database. Standardised operating procedures for analysing these genetic factors were adopted, and criteria for proper nomenclature were developed. Neuroblastoma treatment planning is highly dependant on tumour cell genomic features, and it is likely that a comprehensive panel of DNA-based biomarkers will be used in future risk assignment algorithms applying genome-wide techniques. Consensus on methodology and interpretation is essential for uniform INRG classification and will greatly facilitate international and cooperative clinical and translational research studies.

Modern treatment strategies adjust the therapy of cancer patients according to the predicted biological behaviour of the individual tumour. This is especially important for neuroblastoma patients, as a subset of neuroblastic tumours will undergo spontaneous regression (in infants and in young children) or maturation (in children), whereas others will rapidly progress despite intensive multimodality therapy. This clinical heterogeneity has been known for decades (D’Angio *et al*, 1971; Evans *et al*, 1976). However, patient age and tumour stage alone cannot reliably predict tumour behaviour. Over the past two decades, tumour histology, (Shimada *et al*, 1999) status of the *MYCN* oncogene, (Brodeur *et al*, 1984; Seeger *et al*, 1985), and tumour cell DNA content (ploidy; Look *et al*, 1991; Ladenstein *et al*, 2001) have each been shown to be independently predictive of patient outcome in large retrospective and prospective studies. Besides these two genetic markers, recently 11q aberrations were also included in the INRG pretreatment risk classification (Cohn *et al*, 2009). However, the optimal combination of these and additional recently described prognostic biomarkers to build a treatment stratification algorithm has not been determined yet. Here, we present the consensus of the INRG Biology Committee on which markers should be used currently, on the standardised operating procedures (SOPs; partly on the basis of SIOPEN activities (Ambros *et al*, 2003)) for analysing neuroblastoma tumour molecular diagnostics and genetic nomenclature in this rapidly evolving field.

## Genetic features of neuroblastic tumours associated with favourable clinical behaviour

**Figure 1:**
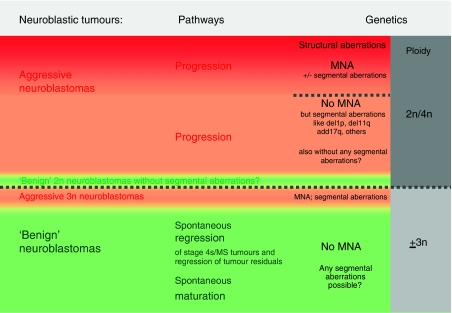
Biological pathways and genetic features in neuroblastic tumours. Tumour cell ploidy (grey columns) can be used to subdivide neuroblastoma tumours into two broad groups (separated by the long punctuated line). Although the ploidy subgroups roughly correspond to the biologic subgroups (aggressive neuroblastomas marked by a red background – either with *MYCN* amplification in dark red and separated by a short punctuated line from neuroblastomas without *MYCN* amplification *vs* less aggressive behaving neuroblastomas indicated by a green background), they do not totally match. Although aggressive near-triploid neuroblastomas (in red below the long punctuated line) have been observed, it is less clear if ‘benign’ diploid neuroblastomas without any structural aberrations (in green above the long punctuated line) occur. ‘Benign’ clinical behaviour refers either to spontaneous regression/maturation without any therapy or with surgery only (no cytotoxic therapy).

Favourable clinical behaviour of neuroblastic tumours is classically characterised by a propensity to undergo spontaneous regression or maturation without cytotoxic therapy (Figure 1). These tumours almost always show whole chromosome gains with few, if any, segmental chromosome aberrations and without gene amplifications. The DNA content is usually in the near-triploid (penta/hexaploid) range (Ambros *et al*, 1995, 1996; Brodeur, 2003; George *et al*, 2007; Mosse *et al*, 2007; Schleiermacher *et al*, 2007). This is also the case for the neuroblastic/ganglionic cell population in Schwann cell stroma-rich tumours, whereas the Schwann cell population itself has a diploid DNA content (Ambros *et al*, 1996; Bourdeaut *et al*, 2008).

Although tumour cell hyperdiploidy (usually near-triploid DNA content) is frequently associated with spontaneous maturation (Ambros *et al*, 1996) and possibly also with spontaneous regression, this ploidy level alone cannot guarantee benign tumour behaviour as near-triploid tumours may also have segmental chromosomal aberrations (i.e., gains or losses of only parts of chromosomes) and *MYCN* amplification, which can lead to clinically aggressive behaviour.

## Genetic features of neuroblastic tumours associated with unfavourable clinical behaviour

Neuroblastomas with an unfavourable clinical behaviour (Figure 1) have a high propensity for locally invasive growth and widespread metastatic dissemination through the lymphatic and haematogenous systems. These tumours frequently show segmental aberrations and high-level amplification of the *MYCN* locus is detected in a substantial subset (Schwab *et al*, 1983; Brodeur *et al*, 1984; Seeger *et al*, 1985). *MYCN* amplification has been shown to be strongly associated with rapid tumour progression and poor prognosis in patients of all ages, with any stage of disease (Brodeur *et al*, 1984; Seeger *et al*, 1985; Ambros *et al*, 1995; Perez *et al*, 2000). As expected, *MYCN* amplification was also highly predictive of worse outcome in the INRG cohort of patients (Cohn *et al*, 2009). Frequently, adjacent or more distantly located genes, such as *DDX1*, *NAG,* and *ALK*, are coamplified with *MYCN,* but amplification events in the absence of *MYCN* amplification are rare. Whether these coamplifications have a prognostic impact awaits clarification (Manohar *et al*, 1995; Squire *et al*, 1995; George *et al*, 1997; De Preter *et al*, 2002; Weber *et al*, 2004).

Although *MYCN* status is central to the risk stratification systems in all cooperative clinical trial groups, it is important to emphasise that the majority of metastatic neuroblastomas do not show amplification of this oncogene (see also Figure 1). Other chromosomal aberrations (and a diploid DNA content) have been assumed to predict unfavourable tumour behaviour, including deletion at the chromosomal region 1p36.3 or 11q23, (Maris *et al*, 1995; Caron *et al*, 1996b; Attiyeh *et al*, 2005) as well as unbalanced gain of the long arm of chromosome 17 (17q21 to 17qter; Caron, 1995; Bown *et al*, 1999; Spitz *et al*, 2003). In addition, some studies have shown that deletions on chromosome 3p, 4p, 9p, and 12p also have prognostic significance (Caron *et al*, 1996a; Thompson *et al*, 2001; Schleiermacher *et al*, 2007). As 11q deletions are inversely associated with *MYCN* amplification, this aberration has emerged as a powerful biomarker of outcome in cases without *MYCN* amplification (Attiyeh *et al*, 2005). Statistical analysis of the INRG database has confirmed this finding, and on the basis of these studies, 11q status has been included as a prognostic criterion in the INRG classification system. (Cohn *et al*, 2009). Recent publications clearly show the potential of comprehensive genome-wide approaches to further refine the prognostic accuracy of somatically acquired chromosomal alterations, (Vandesompele *et al*, 2005; Michels *et al*, 2007; Mosse *et al*, 2007; Schleiermacher *et al*, 2007; Tomioka *et al*, 2008). These studies have shown that in tumours without *MYCN* amplification, segmental chromosome aberrations are associated with clinically aggressive disease. These findings have been extended in a series of 493 *MYCN* amplified and non-amplified neuroblastomas; in tumours with only whole-chromosome copy number variations, there were no disease-related deaths. In contrast, the presence of segmental alterations (see Table 3) with or without *MYCN* amplification was the strongest predictor of relapse (Janoueix-Lerosey *et al*, 2009).

## Tumour cell ploidy

The majority of tumours have a hyperdiploid (near-triploid or penta/hexaploid) DNA content, whereas less than half of neuroblastic tumours are diploid (Look *et al*, 1984; Kaneko *et al*, 1987). Locoregional tumours are commonly hyperdiploid; diploidy is more common in advanced-stage tumours. A number of studies have shown that in patients <18 months of age with metastatic disease, hyperdiploidy in combination with a non-amplified *MYCN* gene and the lack of specific segmental chromosome aberrations (such as 11q deletion) are predictive of a favourable outcome (George *et al*, 2005; Schleiermacher *et al*, 2007). However, in patients <18 months of age with metastatic disease with diploid, *MYCN* non-amplified tumours have a statistically significantly worse outcome.

## Evaluation of genomic markers in the INRG analytic cohort

### Relationship of genetic aberrations

In the INRG analytic cohort (*n*=8800; see Cohn *et al* (2009) for details), 29% were diploid, 16% were *MYCN* amplified, 21% had an 11q aberration, 23% had a 1p aberration, and 48% had a 17q gain. Statistically significant associations of genetic factors were identified (Table 1). 11q aberration was associated with 17q gain (*P*<0.0001) and inversely associated with *MYCN* amplification (*P*=0.0006), but 11q aberration was not associated with diploidy or 1p aberration. Diploidy and 17q gain were not associated, but all other pair-wise comparisons were highly statistically significant (*P*<0.01).

### Outcome by clinical and genetic subgroups

In the survival-tree regression approach of Cohn and Pearson *et al* (2009), patients were clustered into meaningful pretreatment groups that were homogeneous in terms of outcome and prognostic factors (clinical and biological). For the descriptive purposes of this manuscript, the INRG analytic cohort was further subdivided. Outcome by genetic factors *vs* INSS stage, age, and primary tumour site is shown in Tables 2a, and 2b, respectively. Within each genetic factor subgroup, the patterns of outcome differences that have been observed in the overall population prevailed: (a) EFS and overall survival (OS) decreased with increasing stage (Table 2a); (b) older patients had worse outcome than younger patients (Table 2b) and (c) patients with adrenal primary tumour site had worse outcome than those with non-adrenal tumour (Table 2b).

However, as shown in Table 2a, only a limited number of data on the prognostic impact of some segmental aberrations are available, impeding reliable interpretation of some of the data (e.g., 17q gain in stage 1 and 4 s tumours). Thus, larger data sets on segmental aberrations are needed to allow final statements on their prognostic impact (Table 3) .

### Statistical considerations

Tests of association were carried out using a *χ*^2^-test. *P*-values <0.05 were considered statistically significant. For event-free survival (EFS) analysis, time to event was defined as the time from diagnosis until the time of first occurrence of relapse, progression, secondary malignancy, or death, or until time of last contact if no event occurred. For OS, time to event was defined as time until death, or until last contact if the patient was alive. The methods of Kaplan and Meier (1958) were used to calculate EFS and OS estimates, with s.e. according to Peto and Peto (1972).

## International consensus by the INRG biology committee

Because of the relative rarity of neuroblastomas, and the uniqueness of many of the molecular diagnostic factors, the INRG Biology Committee recommended that the genetic studies required for INRG classification be carried out in experienced laboratories, typically central reference laboratories for the cooperative groups (see Table 4), to guarantee high consistency and quality of results (Ambros *et al*, 2003).

### Consensus on the genetic markers to be currently used in the INRG risk classification system

As reported in the study by Cohn and Pearson *et al* (2009) survival-tree regression analyses of the INRG database confirmed the prognostic significance of *MYCN* amplification, 11q aberrations, and ploidy, in addition to age and stage, in different subgroups of the INRG cohort. Table 3 includes these three genetic markers and also those, which should be analysed prospectively.

### Tumour sampling/storing procedure and indication of the tumour cell content

An adequate amount of tumour material (i.e., 10^7^ tumour cells) from at least two different regions of the tumour should be obtained. In collaboration with the institutional pathologist, the tumour cell content must be determined and recorded. The latter is an indispensable prerequisite to avoid false results and has to be carried out by the pathologist. A section (cryo- or paraffin) has to be kept to each tumour piece used for genetic or expression studies. In certain cases, interphase fluorescence *in situ* hybridisation (I-FISH) can be used to decide on the tumour cell number. A tumour cell content of more than 60% is required for most molecular studies and of more than 20% for ploidy measurement. Differentiating/maturing tumours can have fewer tumour cells, and therefore, the interpretation of these data need to de done with caution. For I-FISH, a low tumour cell number can also be sufficient, in case of numeric/structural chromosome aberrations.

Molecular studies on maturing/mature tumours, such as ganglioneuroblastomas or ganglioneuromas, (Ambros *et al*, 1996; Brodeur, 2003) require meticulous identification of tumour and Schwannian stromal cells, and can thus only be undertaken when using an appropriate system (e.g., on ganglionic cells or by microdissection, ideally on paraffin sections). In case of needle-core biopsies, for obtaining tumour material from different areas, an exact determination of the tumour cell content is crucial again (Frostad *et al*, 1999). Interphase fluorescence *in situ* hybridisation results of disseminated tumour cells in the bone marrow (Ambros *et al*, 2001) can be given only if *MYCN* amplification is present due to the higher error-proneness if segmental/numeric aberrations are evaluated in mixtures of normal and tumour cells. In case of immunological preselection and automatic relocation of DTCs (Mehes *et al*, 2001), information on genetic aberrations other than *MYCN* amplification can be given as well.

**Figure 2:**
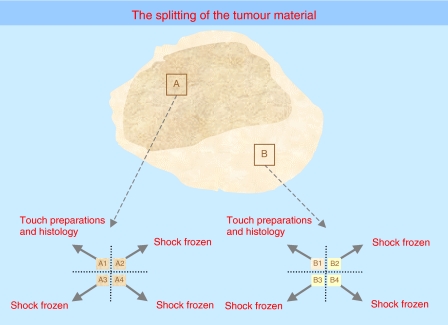
Recommendations concerning the splitting of the tumour material for resected tumours or surgical biopsies. All specimens should be transported to the pathology laboratory as quickly as possible. From there, the snap frozen and/or OCT embedded material should then be transported immediately to the biology lab (can be used for any type of DNA, RNA or protein work). Normal reference cells (e.g., peripheral blood) should be sent to the reference laboratories.

On account of the advent of new techniques enabling large retrospective studies, it is essential to store frozen tumour samples and/or extracted DNA, RNA, or protein (at −80°C or in liquid nitrogen) with exactly determined tumour cell content. Non-neoplastic reference cells from the same patient should be stored as well. The INRG Biology Subcommittee further emphasises the clear need for biobanking of high quality biological materials from neuroblastoma patients, and this must be central to the SOP of any cooperative group for the collection of diagnostic material (Qualman *et al*, 2005). For further details and recommendations, see Figure 2 and the guidelines indicated by Ambros and Ambros (2001).

### Evaluation and the reporting of the *MYCN* gene copy status

**Figure 3:**
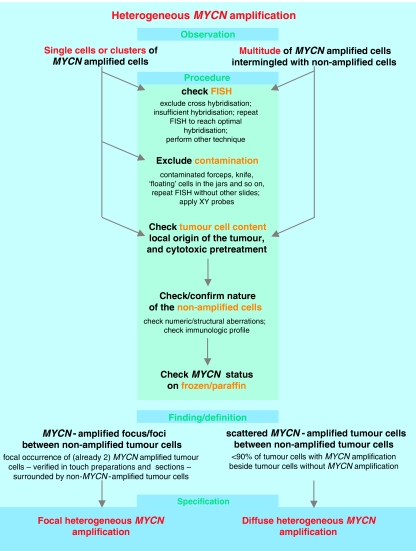
Heterogeneous *MYCN* amplification and recommended procedure for the clarification of the underlying genetic pattern.

The INRG Biology Committee agreed that *MYCN* status should be evaluated in every resected neuroblastic tumour, including the Schwann cell stroma-rich categories (Shimada *et al*, 1999). Recommended and accepted techniques to detect structural and/or segmental aberrations are summarised in Table 3, and recommendations concerning the evaluation and terminology are given in Tables 5, 6, 7 and 8. Interphase fluorescence *in situ* hybridisation is preferred because it has a number of advantages compared with the other techniques, most important of which is direct quality control of the hybridization quality by the microscopist. Heterogeneous *MYCN* amplification requires meticulous analysis of the tumour specimen (Figure 3). At the present time, the prognostic significance of heterogeneously *MYCN* amplified neuroblastomas is not known, but amplification of a substantial number of tumour cells in a specimen is still considered an ominous sign.

### Evaluation and reporting of segmental genetic aberrations: gains and losses of chromosome parts

The term ‘structural’ aberrations (also known as structural chromosomal instability (s)-CIN) designates any kind of chromosomal alterations including gene amplification. To produce precise definitions, consensus was reached to introduce the term ‘segmental’ aberrations for gains and losses of chromosome parts. In neuroblastomas, most segmental chromosome aberrations are unbalanced, that is, they are associated with regional losses or gains of chromosome parts. Balanced aberrations, that is, reciprocal translocations without losses of genetic material, are thought to be relatively uncommon in neuroblastomas. Segmental aberrations (such as 11q23 and 1p36.3 deletions) can be identified by a number of techniques shown in Table 3. Currently, either I-FISH or PCR are carried out to detect segmental aberrations, but the INRG Biology Committee recommends array-based methods, multiplex ligation-dependent probe amplification (MLPA) or similar techniques in the future. The latter techniques are currently being validated (for nomenclature (I-FISH, PCR, and MLPA), see Tables 5, 6, 7 and 8; part of the nomenclature was developed by the SIOPEN Biology Group (for group members, see Table 4 and Ambros *et al*, 2003)).

### Tumour cell DNA content (ploidy)

Ploidy was analysed in the INRG cohort using the definitions diploid (i.e., DI ⩽1.0) *vs* hyperdiploid (DI >1.0) as published in Look *et al* (1991). More recent data suggest that prognostic classification may be further refined using specific ranges of DI The INRG Biology Committee recommends recording the exact numerical DI value for each tumour, so that clinically relevant cutoffs for DI can be determined (for techniques see Table 3). Any other method can be applied as long as it allows discrimination of the diploid DNA peak. However, an unknown number of normal cells contained in the tumour specimen under investigation, including tumour-derived, but non-neoplastic diploid Schwann cells are an important source of false data interpretation. Thus, the DNA content of maturing/mature tumours, which mostly develop from near-triploid neuroblastomas is easily misdiagnosed as diploid.

## Future prospects: new techniques

There is clear consensus that the use of genetic data derived from diagnostic neuroblastoma tumours will remain central to patient treatment planning and gain even more power. The INRG Biology Committee addressed the issues of ongoing and future work directed at prognostic biomarker discovery and validation, as well as how these data sets can be leveraged to identify molecular targets for novel therapeutics. In Table 3, 12 genetic markers to be analysed prospectively are mentioned. Pan-genomic investigations may help to identify additional genomic areas of interest.

### DNA-based biomarkers

A number of studies suggest that the pattern of DNA-based genomic changes is prognostic in neuroblastoma, and that whole genome analysis should be carried out rather than a series of individual locus-specific assays. Therefore, the INRG Biology Subcommittee suggests using pan- or multigenomic techniques enabling an analysis of all relevant genomic loci.

Multiplex ligation-dependent probe amplification is a PCR-based technique that detects a large variety of segmental aberrations and gene amplifications in a robust manner (for technical details, see (Schouten *et al*, 2002). Quantification of losses, gains, and amplifications in small amounts of DNA can be determined, and simultaneous investigation of a large number of loci, covering all currently known important aberrant regions found in neuroblastomas, can be carried out in a single assay (Villamon *et al*, 2008). The robust nature of the results and the relatively low cost of the MLPA kits make this technique attractive for routine neuroblastoma analysis.

Chip-based technologies have also been used to molecularly classify neuroblastoma tumours (Bilke *et al*, 2005; George *et al*, 2005; Selzer *et al*, 2005; Spitz *et al*, 2006; Stallings *et al*, 2006; Lastowska *et al*, 2007; Michels *et al*, 2007; Mosse *et al*, 2007; Tomioka *et al*, 2008). Comparative genomic hybridisation has reached a high coverage of the target sequences and become more widely used. The advantage of single nucleotide polymorphism (SNP)-based platforms is the simultaneous detection of physical gains and losses and the detection of copy neutral LOH (George *et al*, 2005). Still, these techniques cannot be considered as routine techniques but as excellent tools to identify so far undetected genomic regions of prognostic impact. The INRG Biology Committee considers it a priority to work towards a single diagnostic tool that will reliably and accurately detect allelic deletions and gains, as well as *MYCN* amplification, and estimate overall DNA content (ploidy), on a single platform. This has been achieved using in-house arrays (Maris *et al*, 2005; Janoueix-Lerosey *et al*, 2009), but a commercially available platform will be needed to implement this or a similar technology (e.g., MLPA) uniformly in clinical laboratories across various groups and countries.

### RNA-based biomarkers

Several groups have recently shown that genome-wide gene expression profiling can identify differentially expressed transcripts that provide reliable prognostic information (Wei *et al*, 2004; Ohira *et al*, 2005; Schramm *et al*, 2005; Asgharzadeh *et al*, 2006; Oberthuer *et al*, 2006; Lastowska *et al*, 2007; Tomioka *et al*, 2008). Although these studies are of outstanding quality, they each suffer from relatively small sample sizes, necessitating preliminary validation of proposed gene sets. In addition, there was very little overlap in the gene sets that were identified as prognostic by each group, so a consensus gene set, that is, predictive of outcome has yet to be identified. International collaboration will be required to unequivocally determine whether mRNA expression profiles, likely focused on a representative set of genes, are a sensitive and specific enough molecular assay to be used in the clinic, and/or if these data are synergistic with, or override the information derived from the assays of DNA alterations.

### Molecular targets

Neuroblastoma treatment will continue to rely on risk grouping based on tumour genomic features. Increasing attention is now focused on utilising these data sets to discover therapeutic targets. It stands to reason that any prognostically relevant genomic aberration might also signal a molecular aberration that is critical to the maintenance of the malignant phenotype, and thus can be targeted for therapy. Regions of DNA copy number gain that result in the overexpression of a protein that is druggable in patients succumbing to the disease can theoretically be identified in carefully annotated data sets in which both high quality DNA- and RNA-based microarray data are available. Thus, a parallel focus on therapeutic target discovery and validation will further increase the significance of genomic efforts used in the discovery, validation, or even clinical application phases of implementation.

## Conclusion

Currently, neuroblastoma treatment planning is not possible without detailed knowledge of tumour cell genomics. International efforts were and are needed not only to identify the most significant genetic markers but also to identify and coordinate particularly suitable techniques for their evaluation in all patients. For future studies, new technologies, which provide a comprehensive picture of the tumour cell genome are recommended. Moreover, the INRG Biology Committee achieved consensus on the nomenclature of genetic aberrations and developed definitions of the terms to be used. The INRG Biology Committee is dedicated to providing the highest possible reproducibility and reliability of genetic markers enabling a uniform INRG classification and forming the basis for international clinical and translational studies. Finally, many of these recommendations apply not only to neuroblastoma tumours but also to any tumour entity for which genetic factors are essential for therapy decisions.

## Figures and Tables

**Table 1:** Tests of association of genetic factors

	***MYCN* amplification**	**1p aberration**	**11q aberration**	**17q gain**
Diploidy	*P*<0.0001	*P*<0.0001	*P*=0.1242	*P*=0.3613
*MNA*		*P*<0.0001	*P*=0.0006^*^	*P*=0.0096
1p aberration			*P*=0.0613	*P*<0.0001
11q aberration				*P*<0.0001

^*^Inversely associated. MNA *MYCN* amplification

**Table 2a:** Event-free and overall survival (EFS and OS) of genetic factors by INSS stage

	**INSS stage 1**			**INSS stage 2**			**INSS stage 3**			**INSS stage 4s**			**INSS Stage 1,2,3,4s**			**INSS Stage 4**		
	***n* (%)**	**5-year EFS±s.e.**	**5-year OS±s.e.**	***n* (%)**	**5-year EFS±s.e.**	**5-year OS±s.e.**	***n* (%)**	**5-year EFS±s.e.**	**5-year OS±s.e.**	***n* (%)**	**5-year EFS±s.e.**	**5-year OS±s.e.**	***n* (%)**	**5-year EFS±s.e.**	**5-year OS±s.e.**	***n* (%)**	**5-year EFS±s.e.**	**5-year OS±s.e.**
Hyper diploid (*n*=2611)	719 (28)	93±2	99±1	415 (16)	88±3	96±2	452 (17)	82±3	86±3	232 (9)	86±3	92±3	1818 (70)	88±1	94±1	728 (28)	47±3	53±3
Diploid *n*=1086)	196 (18)	90±4	96±2	119 (11)	84±5	90±5	152 (14)	65±6	66±6	62 (6)	77±9	77±9	529 (49)	80±3	84±3	527 (50)	31±3	36±3
*MYCN,* not amplified (*n*=5947)	1626 (27)	93±1	98±1	947 (16)	85±2	97±1	1013 (17)	81±2	89±1	481 (8)	82±2	91±2	4067 (68)	87±1	95±1	1798 (31)	45±2	54±2
*MYCN,* amplified (*n*=1155)	48 (4)	50±12	76±9	39 (3)	57±12	67±11	217 (19)	45±4	48±4	47 (4)	41±9	45±9	351 (30)	46±4	53±4	787 (69)	21±2	25±2
1p normal (*n*=1659)	489 (29)	93±2	98±1	264 (16)	83±3	98±1	313 (19)	77±3	88±3	152 (9)	86±4	95±3	1218 (73)	86±1	95±1	430 (26)	42±3	50±3
1p aberration (*n*=493)	48 (10)	76±9	100	29 (6)	63±11	81±9	90 (18)	52±7	61±7	29 (6)	60±13	67±12	196 (40)	60±5	74±4	280 (57)	24±4	32±4
11q normal (*n*=844)	227 (27)	90±3	99±1	141 (17)	71±7	95±3	152 (18)	77±5	85±4	69 (8)	86±7	95±5	589 (70)	82±3	94±2	233 (28)	42±5	50±5
11q aberration (*n*=220)	11 (5)	73±22	91±14	11 (5)	45±34	91±16	35 (16)	51±11	75±11	8 (4)	38±30	63±38	65 (30)	51±10	79±8	153 (70)	28±6	47±7
17q normal (*n*=187)	36 (19)	78±9	97±4	22 (12)	77±12	100	47 (25)	74±7	85±6	15 (8)	92±11	92±11	120 (64)	78±5	92±3	60 (32)	40±7	46±7
17q gain (*n*=175)	20 (11)	95±9	100	19 (11)	51±18	84±14	16 (9)	75±13	94±7	4 (2)	100	100	59 (34)	76±8	93±5	104 (59)	24±6	38±7

**Table 2b:** Event-free and overall survival (EFS and OS) of genetic factors, by age and primary tumour site at diagnosis

	**Age <547 days**			**Age > 547 days**			**Adrenal primary tumour site**			**Non-adrenal primary tumour site**		
	***n* (%)**	**5-year EFS±s.e.**	**5-year OS±s.e.**	***n* (%)**	**5-year EFS±s.e.**	**5-year OS±s.e.**	***n* (%)**	**5-year EFS±s.e.**	**5-year OS±s.e.**	***n* (%)**	**5-year EFS±s.e.**	**5-year OS±s.e.**
Hyper diploid (*n*=2611)	1816 (70)	88±1	95±1	795 (30)	48±3	54±3	1023 (39)	73±2	78±2	1473 (56)	78±2	85±1
Diploid (*n*=1086)	425 (39)	70±4	75±4	661 (61)	46±3	50±3	489 (45)	49±4	54±3	556 (51)	60±3	64±3
*MYCN,* not amplified (*n*=5947)	3657 (61)	88±1	95±1	2290 (39)	51±1	60±1	2439 (41)	69±1	76±1	3265 (55)	77±1	86±1
*MYCN,* amplified (*n*=1155)	404 (35)	36±3	41±3	751 (65)	26±2	30±2	732 (63)	27±2	33±2	384 (33)	33±4	37±4
1p normal (*n*=1659)	1095 (66)	88±1	96±1	564 (34)	49±3	59±3	751 (45)	71±2	78±2	881 (53)	77±2	88±2
1p aberration (*n*=493)	185 (38)	57±5	65±5	308 (62)	26±3	38±4	323 (66)	33±4	44±4	157 (32)	48±5	58±5
11q normal (*n*=844)	510 (60)	83±3	93±2	334 (40)	46±4	57±4	359 (43)	64±4	72±4	451 (53)	73±3	85±3
11q aberration (*n*=220)	63 (29)	61±11	85±8	157 (71)	26±5	47±6	126 (57)	34±6	52±7	93 (42)	35±9	63±9
17q normal (*n*=187)	104 (56)	80±5	92±3	83 (44)	44±6	53±6	74 (40)	60±7	65±7	105 (56)	67±6	82±5
17q gain (*n*=175)	65 (37)	68±9	83±7	110 (63)	27±5	41±6	116 (66)	37±7	51±7	53 (30)	53±9	67±9

**Table 3:** Consensus of genetic markers currently used for therapy stratification and proposed for future analyses

**Genetic INRG risk classification markers**	**Techniques recommended/accepted**	**DNA probes recommended and comments**
**Obligatory markers**		
*MYCN*	I-FISH PCR, aCGH, MLPA	Two colour I-FISH: BAC or other large DNA-insert clone for the *MYCN* gene and a clone of similar size for a gene/locus on the long arm of chromosome 2 (e.g., LAF at 2q11); commercially available probes should be used whenever possible
11q23	I-FISH, PCR; pan-/multigenomic techniques: aCGH (oligo, clone or SNP based); MLPA	
Ploidy	Flow or static cytometry	Normal cells from the same patient should be used as a diploid standard
		
**Genetic markers to be analysed prospectively**		
1p	aCGH, SNP arrays, MLPA etc.	Commercially available platform preferred
2p		
*DDX1*		
*NAG*		
*ALK*		
3p		
4p		
7q		
9p		
12p		
14q		
17q		
Others	aCGH, SNP arrays	

aCGH=array-based comparative genomic hybridisation; BAC=bacterial artificial chromosomes; I-FISH=interphase fluorescence *in situ* hybridisation; INRG=International Neuroblastoma Risk Group; MLPA=multiplex ligation-dependent probe amplification; PCR=polymerase chain reaction; SNP=single nucleotide polymorphism.

**Table 4:** Laboratories responsible for Neuroblastom Genetics

**National Group**	**Name**		**Institution**	**Town**	**Country**
Australia	Michelle	Haber, PhD	Children's Cancer Institute Australia and COG	Sydney, NSW	Australia
COG	Julie	Gastier-Foster, PhD	Nationwide Children's Hospital	Columbus, OH	USA
	Michael	Hogarty, MD	Children's Hospital of Philadelphia	Philadelphia, PA	USA
	Rochelle	Bagatell, MD	Children's Hospital of Philadelphia	Philadelphia, PA	USA
Germany	Frank	Berthold, MD	Department of Pediatric Hematology and Oncology, University of Cologne	Cologne	Germany
	Jessica	Theißen, PhD	Department of Pediatric Hematology and Oncology, University of Cologne	Cologne	Germany
	Manfred	Schwab, PhD	Division of Cytogenetics, German Cancer Research Center	Heidelberg	Germany
	Frank	Westermann, MD	Division of Cytogenetics, German Cancer Research Center	Heidelberg	Germany
	Freimut H	Schilling, MD	Department of Pediatric Oncology and Hematology, Olgahospital	Stuttgart	Germany
	Sabine	Stegmaier, PhD	Department of Pediatric Oncology and Hematology, Olgahospital	Stuttgart	Germany
	Niggli	Felix, MD	Department of Pediatric Oncology and Hematology	Zurich	Switzerland
Japan	Akira	Nakagawara, MD	Chiba Cancer Center Research Institute	Chiba	Japan
	Miki	Ohira, MD	Chiba Cancer Center Research Institute	Chiba	Japan
	Yasuhiko	Kaneko, MD	Saitama Cancer Center Research Institute	Saitama	Japan
	Junko	Takita, MD	Department of Pediatrics, University of Tokyo	Tokyo	Japan
	Hajime	Ohkita, MD	National Center for Child Health and Development	Tokyo	Japan
SIOPEN Biology	Peter F	Ambros, PhD	CCRI, Children's Cancer Research Institute	Vienna	Austria
	Inge M	Ambros, MD	CCRI, Children's Cancer Research Institute	Vienna	Austria
	Frank	Spelemann, PhD	Center for Medical Genetics-OK5, University of Ghent	Ghent	Belgium
	Nadine	Van Roy, PhD	Center for Medical Genetics-OK5, University of Ghent	Ghent	Belgium
	Ales	Vicha, MD	Department of Pediatric Hematology and Oncology, Motol	Prague	Czech Republic
	Jean	Bénard, MD	Lab De Pharmacologie Clinique et Moleculaire, Instiut Gustav Roussy	Villejuife-Cedex	France
	Alexander	Valent, PhD	Lab De Pharmacologie Clinique et Moleculaire, Instiut Gustav Roussy	Villejuife-Cedex	France
	Jérome	Couturier, MD	Unité de Cytogénétique oncologique, Institut Curie – Section Médicale	Paris	France
	Olivier	Delattre, PhD	Unité de Cytogénétique oncologique, Institut Curie – Section Médicale	Paris	France
	Gudrun	Schleiermacher, MD	Unité de Cytogénétique oncologique, Institut Curie, Section Médicale	Paris	France
	Marta	Jeison, PhD	Pediatric Hematology Oncology Department, Schneider Children's Medical Center of Israel	Petah Tikva	Israel
	Gian Paolo	Tonini, PhD	Translational Paediatric Oncology, National Cancer Research Institute (IST)	Genoa	Italy
	Raffaella	Defferrari, PhD	Translational Paediatric Oncology, National Cancer Research Institute (IST)	Genoa	Italy
	Katia	Mazzocco, PhD	Translational Paediatric Oncology, National Cancer Research Institute (IST)	Genoa	Italy
	Klaus	Beiske, MD	Department of Pathology, Rikshospitalet	Oslo	Norway
	Barbara	Marques, PhD	Centro de Genética Humana, Instituto Nacinal de Saúde	Lisbon	Portugal
	Nicole	Gross Ph.D.	Recherche en Oncologie Pédiatrique	Lausanne	Switzerland
	Rosa	Noguera, MD	Facultad de Medicina	Valencia	Spain
	Tommy	Martinsson, PhD	Gahlgrenska Univ. Hospital/East, Gothenburg University	Gothenburg	Sweden
	Deborah A.	Tweddle, MD	Department of Health	Newcastle	UK
	John	Lunec, PhD	Northern Institute for Cancer Research	Newcastle	UK
	Nick	Bown, PhD	Institute of Human Genetics	Newcastle	UK

**Table 5:** *MYCN* terms and definitions of I-FISH results

***MYCN* status analysed by I-FISH**		
**Terms**	**Description**	**Comments**
*MYCN* not amplified (normal *MYCN* status)	A balanced ratio between the *MYCN*-specific signals and the signal number of the reference probe on the chromosome 2q arm	Neuroblastic tumours have a propensity to polyploidisation, especially but not exclusively after therapy with the occurrence of giant polyploid nuclei. The number of *MYCN* signals must not exceed the number of reference signals on 2q (caveat: the centromeric probe is unqualified as reference probe because of frequent centromeric associations in such tumours). The number of other chromosomes or the DNA index should be checked as well.
2p24 gain:	The term 2p24 gain is suggested as a descriptive generic term for *MYCN* signal numbers exceeding up to 4-fold the number of reference signals on chromosomal arm 2q. This pattern could reflect either:	The use of a 2p specific probe in addition to 2q (optionally a centromeric probe) is recommended to clarify the presence of a chromosome 2p gain *vs* a restricted *MYCN* gain. The discrimination could be important since a *MYCN* gain could indicate an ‘incipient’ *MYCN* amplification.
(a) 2p gain	(a) a gain of short arms of chromosome 2, for example, from unbalanced translocations involving 2p; or	Equal number of *MYCN* and 2p signals, exceeding 2q signals (mostly a consistent excess of one or two *MYCN* and 2p signals).
(b) *MYCN* gain	(b) a gain of the *MYCN* gene either chromosomally integrated (such as duplications) or extrachromosomally	An inconsistent *MYCN* excess with a varying number of excess signals is much more likely in line with *MYCN* gain as extrachromosomally elements. The latter can also be found in tumours with heterogeneous *MYCN* amplification.
*MYCN* amplification, ‘homogeneous’	More than 4-fold increase in the *MYCN* signal number compared with the reference probe located on the chromosome 2q	Details on the amplification grade (a) >4–10 times; (b) >10 times; (c) >30 times amplification, and also on the type of amplification either double minutes (dmin) or homogeneously staining regions (hsr) should be given in the report. Besides tumour cells with amplification defined as such, a proportion of tumour cells can also show ‘*MYCN* gain’. However, tumour cells without *MYCN* gain or amplification are extremely rare.
*MYCN* amplification, ‘heterogeneous’	Generic term for the coexistence of amplified as well as non-amplified tumour cells in the same tumour	Single cells (at least two) or cell clusters or a multitude of cells with MNA besides proven tumour cells without MNA. The terms ‘focal’ and ‘diffuse MNA’ are specifications, which, however, can only be attributed after evaluation of the tissue sections. In case amplified tumour cells are found in addition to non-amplified cells, a very meticulous procedure is strongly recommended to exclude false-positive or false negative results (see Figure 3).
(a) Focal heterogeneous *MYCN* amplification	Defined as the more or less focal (one focus or several foci – multifocal) occurrence of *MYCN*-amplified cells surrounded by non-amplified tumour cells.	
(b) Diffuse heterogeneous *MYCN* amplification	Designates a tumour, which contains the *MYCN* amplified cells in a scattered pattern besides non-*MYCN* amplified tumour cells.	
No result	Should be specified: unclear or not interpretable;	
	inadequate tumour cell content; no tumour; not carried out	

I-FISH=interphase fluorescence *in situ* hybridisation.

**Table 6:** Terms and definitions for segmental chromosome aberrations of I-FISH results

**Segmental chromosome aberrations analysed by I-FISH**		
**Terms**	**Description**	**Comments**
Normal status	Balanced ratio between the signal numbers of the chromosomal region of interest and the reference signals on the opposite arm of the chromosome	In the case of a 2/2 ratio, an isodisomy with a complete LOH of all loci located on the investigated chromosome cannot be excluded. However, uniparental isodisomies are extremely rare in NB tumours.
I-FISH imbalance (inconclusive with regard to LOH)	Unbalanced ratio between the signal numbers of the chromosomal region of interest and the reference signals with more than one signals of the chromosomal region of interest	Does not necessarily correspond to an LOH, and is therefore called inconclusive. PCR should be performed to clarify this result. In NB tumours, imbalances frequently, but not always, reflect LOH. Fluorescence *in situ* hybridisation imbalances can also occur focally. Interphase fluorescence *in situ* hybridisation imbalances should only be diagnosed if hybridisation failure can be excluded. At least 200 cells should be evaluated.
Deletion	Unbalanced ratio between the signal numbers of the chromosomal region of interest and the reference signals with only one signal of the chromosomal region of interest	This hybridisation pattern corresponds to an LOH. Deletions can also occur focally. Interphase fluorescence *in situ* hybridisation deletion should only be diagnosed if hybridisation failure can be excluded. At least 200 cells should be evaluated.
Gain	Up to 4-fold excess of signal numbers of the chromosomal region of interest compared with the reference signals	
No result	Should be specified: unclear or not interpretable; inadequate tumour cell content; no tumour; Not carried out	

I-FISH=interphase fluorescence *in situ* hybridisation.

**Table 7:** Terms and definitions of segmental chromosome aberrations analysed by PCR

**Losses of chromosomal parts analysed by PCR**		
**Terms**	**Description**	**Comments**
**Normal status**	**Similar band intensities**	
Allelic imbalance (inconclusive)	Different band intensities; The result can mean either allele disequilibrium or LOH	One band is relatively weaker, when compared with the ratio of constitutional DNA controls. Needs further clarification by FISH (copy number of the respective chromosome has to be known) and re-evaluation of the tumour cell content (an LOH could be masked by a high number of normal cells). Alternatively, different PCR probes, located on both arms (p and q) can circumvent the need for the use of another technique. Allele disequilibrium means an imbalance between the number of maternal and paternal alleles (in the case of odd numbers of the respective chromosome), but not a physical loss of alleles
Allelic loss	Loss of one band	One band has completely, or almost completely disappeared
No result	Should be specified: unclear or not interpretable; inadequate tumour cell content (<60%); constitutional homozygosity; no DNA; no tumour; not carried out	

FISH=fluorescence *in situ* hybridisation.

**Table 8:** Terms and definitions of chromosome aberrations analysed by MLPA

**Chromosome and gene status analysed by MLPA**		
**Terms**	**Description**	**Comments**
Normal status	Balanced ratio between the majority of signals (signal intensity is visualised in the graphic representation of the MLPA results as height of the bars) of both chromosomal arms	In the case of a balanced ratio, a uniparental isodisomy (a uniparental isodisomy means that both chromosomes are derived from one parent. Thus, the two chromosomes are not homologous but identical. However, uniparental isodisomies are rare in NB tumours) with a complete LOH of all loci located on the investigated chromosome cannot be excluded
*Segmental chromosome aberration*		
Loss	Unbalanced ratio between the signals of the chromosomal region of interest (at least two adjacent probes) and the reference signals (at least two probes) signals	This result could correspond to a FISH deletion that reflects an LOH, or a FISH imbalance, which does not necessarily indicate an LOH.
Gain	Unbalanced ratio (low signal excess) between the signals of the chromosomal region of interest (at least two adjacent probes) and the reference signals (at least two) of the chromosomal region of interest	
	The threshold between gain and amplification needs to be determined for the experimental system with the help of other techniques,for example, I-FISH	
Amplification	Unbalanced ratio (high signal excess) between the signals of a gene and all other probes located on the same chromosome	The threshold between gain and amplification needs to be determined for the experimental system with the help of other techniques, for example, I-FISH
No result	Should be specified: unclear or not interpretable; inadequate tumour cell content; no tumour; not carried out	

FISH=fluorescence *in situ* hybridisation; MLPA=multiplex ligation-dependent probe amplification; I-FISH=interphase fluorescence *in situ* hybridisation.
